# IMMUNOGENICITY AND IMPACT ON NASOPHARYNGEAL CARRIAGE OF A SINGLE DOSE OF PCV10 GIVEN TO VIETNAMESE CHILDREN AT 18 MONTHS OF AGE

**DOI:** 10.1016/j.lanwpc.2021.100273

**Published:** 2021-09-20

**Authors:** Rachel A Higgins, Beth Temple, Vo Thi Trang Dai, Thanh V Phan, Nguyen Trong Toan, Leena Spry, Zheng Quan Toh, Monica L Nation, Belinda D Ortika, Doan Y Uyen, Yin Bun Cheung, Cattram D Nguyen, Kathryn Bright, Jason Hinds, Anne Balloch, Heidi Smith-Vaughan, Tran Ngoc Huu, Kim Mulholland, Catherine Satzke, Paul V Licciardi

**Affiliations:** 1 Infection and Immunity, Murdoch Children's Research Institute, Melbourne, Australia; 2 Department of Paediatrics, The University of Melbourne, Melbourne, Australia; 3 Global Health, Menzies School of Health Research, Charles Darwin University, Darwin, NT, Australia; 4 Epidemiology and Population Health, London School of Hygiene & Tropical Medicine, London, UK; 5 Microbiology and Immunology, Pasteur Institute of Ho Chi Minh City, Ho Chi Minh City, Vietnam; 6 Department of Disease Control and Prevention, Pasteur Institute of Ho Chi Minh City, Ho Chi Minh City, Vietnam; 7 Centre for Quantitative Medicine, Duke-NUS Medical School, Singapore; 8 Centre for Child Health Research, University of Tampere and Tampere University Hospital, Tampere, Finland; 9 Institute for Infection and Immunity, St George's, University of London, London, UK; 10 BUGS Bioscience, London Bioscience Innovation Centre, London, UK; 11 Department of Microbiology and Immunology at the Peter Doherty Institute for Infection and Immunity, The University of Melbourne, Australia

## Abstract

**Background:**

This study investigated the immunogenicity and impact on nasopharyngeal carriage of a single dose of PCV10 given to 18-month-old Vietnamese children. This information is important for countries considering catch-up vaccination during PCV introduction and in the context of vaccination during humanitarian crises.

**Methods:**

Two groups of PCV-naïve children within the Vietnam Pneumococcal Project received PCV10 (n=197) or no PCV (unvaccinated; n=199) at 18 months of age. Blood samples were collected at 18, 19, and 24 months of age, and nasopharyngeal swabs at 18 and 24 months of age. Immunogenicity was assessed by measuring serotype-specific IgG, opsonophagocytosis (OPA) and memory B cells (Bmem). Pneumococci were detected and quantified using real-time PCR and serotyped by microarray.

**Findings:**

At 19 months of age, IgG and OPA responses were higher in the PCV10 group compared with the unvaccinated group for all PCV10 serotypes and cross-reactive serotypes 6A and 19A. This was sustained out to 24 months of age, at which point PCV10-type carriage was 60% lower in the PCV10 group than the unvaccinated group. Bmem levels increased between 18 and 24 months of age in the vaccinated group.

**Interpretation:**

We demonstrate strong protective immune responses in vaccinees following a single dose of PCV10 at 18 months of age, and a potential impact on herd protection through a substantial reduction in vaccine-type carriage. A single dose of PCV10 in the second year of life could be considered as part of catch-up campaigns or in humanitarian crises to protect children at high-risk of pneumococcal disease.

## 1 Evidence before this study

Infant vaccination with pneumococcal conjugate vaccine (PCV) is a proven strategy to reduce vaccine-type pneumococcal carriage and disease. Infant PCV introduction may or may not be coupled with catch-up vaccination in older children. Modelling studies from Vietnam and Kenya suggest that the addition of catch-up vaccination to infant PCV introduction accelerates both direct and indirect protection effects. PCV vaccination could also be an important strategy to protect older children affected by humanitarian crises and other emergency settings. The World Health Organization (WHO) currently recommends PCV catch-up vaccination campaigns in older children but notes that there is a lack of data to determine whether one or two doses should be given during the second year of life. We searched PubMed to 28^th^ February 2021 using search terms including but not limited to “pneumococcal conjugate vaccine”, “catch-up vaccination”, “toddler”, “older children”, “humanitarian”, “immunogenicity” and “carriage”. Three trials report immunogenicity data after one dose of PCV in the second year of life: two using PCV13 and one using PCV10. A trial from Burkina Faso and a multicentre meningococcal vaccine trial both found that a single dose of PCV13 among children aged 12-15 months was immunogenic for most serotypes. A trial from Kenya found that a single dose of PCV10 among children 1-4 years of age reduced vaccine-type pneumococcal carriage and showed good immunogenicity, noting however that immune responses were lower among children aged 12-23 months than those aged 24-59 months for five serotypes. More data are needed to facilitate decision-making on optimal catch-up campaigns as part of infant PCV vaccine introduction as well as formulating recommendations for PCV use during humanitarian crises.

## 2 Added value of this study

This study is the first from Asia to evaluate a single PCV10 dose in the second year of life, and the first study providing vaccination at 18 months of age. We undertook a comprehensive assessment of PCV10 immunogenicity including serotype-specific IgG and opsonophagocytic assays (OPA) to all ten vaccine serotypes as well as the cross-reactive serotypes 6A and 19A. We also measured serotype-specific memory B cell responses (Bmem) to several serotypes as an indicator of long-term protection. Nasopharyngeal carriage of pneumococcal bacteria was measured by qPCR combined with molecular serotyping by microarray. Using this approach, we demonstrated that a single dose of PCV10 at 18 months of age produced robust immunogenicity in terms of IgG and OPA responses for all vaccine serotypes compared with unvaccinated children that persisted for six months. Bmem levels were higher after six months for two serotypes (1 and 18C). By 24 months of age, there was a 60% reduction in vaccine-type pneumococcal carriage. We also show a disconnect between IgG and OPA responses for serotypes 1, 5 and 6B after a single PCV10 dose; serotypes 1 and 5 are common causes of invasive pneumococcal disease, particularly in Africa, while serotype 6B is a common carriage serotype globally. OPA is regarded as a better marker of protection.

## 3 Implications of all the available evidence

Results from this study are consistent with previous data from other parts of the world and support the use of a single dose of PCV10 as part of catch-up campaigns associated with infant PCV vaccination as well as for campaigns in high-risk settings such as humanitarian crises. Our data suggest that protection will extend beyond the second year of life, covering a time when risk of infection and particularly transmission are high. In regions where serotypes 1 and 5 are dominant, a second dose might be considered to improve the disconnect between IgG and OPA responses, as has been reported previously. This information will be important for countries considering catch-up campaigns as part of PCV introduction as well as informing recommendations for the use of PCV in emergency settings.

## 4 INTRODUCTION

*Streptococcus pneumoniae* (the pneumococcus) is a leading cause of mortality and morbidity in children less than five years of age worldwide.[1] The introduction of pneumococcal conjugate vaccines (PCVs) into routine infant immunisation schedules has led to a decline in vaccine-type (VT) pneumococcal disease.[2], [3], [4] PCVs reduce pneumococcal disease by eliciting serotype-specific antibody responses and by reducing VT nasopharyngeal carriage, which leads to reduced pneumococcal transmission and herd protection in the broader population. Modelling studies from Vietnam[5] and Kenya[6] suggest that coupling infant PCV introduction with catch-up vaccination among older children accelerates both direct and indirect protection, but there is limited empirical evidence to support this. The World Health Organization (WHO) recommends the use of catch-up vaccination at the time of PCV introduction wherever possible, noting that there are currently not enough data to determine whether two doses are required for children aged 12 to 23 months, as recommended by the manufacturers, or whether one dose is sufficient.[7]

Only three trials have reported immunogenicity data following a single dose of PCV in the second year of life. Strong immune responses were seen three months after a dose of 13-valent PCV (PCV13; *Prevnar-13*®, Pfizer) administered at 12-15 months of age in Burkina Faso[8], and one month after a dose administered at 12-14 months of age in a multicentre meningococcal vaccine trial.[9] A trial of ten-valent PCV (PCV10; *Synflorix* ®, GlaxoSmithKline Biologicals) among children aged 1-4 years in Kenya showed that a single dose provided good immunogenicity one-month post-vaccination and also reduced vaccine-type pneumococcal carriage two-months post-vaccination.[10]

PCV vaccination in older children could also be an important public health tool for populations affected by humanitarian crises and in other emergency settings, particularly famine where malnourished children are at very high risk of death from pneumonia.[11,12] WHO recommends that, during emergency situations, PCV is used in children under one and considered for use in children under five, although there are no clear guidelines on the target age, number of doses or frequency of campaigns due to a lack of evidence.[13,14] The COVID-19 pandemic has profoundly affected nutrition and vaccine access for the poorest children in the world[15,16]. Whilst nonpharmaceutical interventions introduced during the pandemic may reduce transmission of other respiratory pathogens[17], post-pandemic there is likely to be a large population of increasingly vulnerable, unvaccinated children at high risk of pneumonia. A single dose of PCV to high-risk children 18 months and older in the most difficult of settings has the potential to protect those children and their communities.

A single dose of PCV during the second year of life could accelerate the impact of PCV introduction and could provide a feasible and cost-effective approach to protect vulnerable populations during emergency situations. However, immunogenicity and carriage data to support this approach are limited. In Vietnam, PCV is not currently part of the routine infant immunisation program, enabling evaluation of a single dose approach among unvaccinated children. We assessed the immunogenicity and impact on carriage of a single dose of PCV10 at 18 months of age as part of a larger trial of different infant pneumococcal vaccination schedules in Vietnamese children. We previously reported that pneumococcal carriage among unvaccinated participants peaked at 24.5% at 12 months of age with a similar prevalence of 23.9% at 18 months of age,[18] suggesting the value of strategies to protect children during the second year of life. We measured immune responses including serotype-specific IgG, opsonophagocytosis and memory B cells (Bmem) in vaccinated and unvaccinated children, as well as pneumococcal carriage rates and density.

## 5 METHODS

### 5.1 Study design

This study was part of the Vietnam Pneumococcal Project, the protocol for which has been published previously.[14] The study was approved by the Institutional Review Board at the Pasteur Institute of Ho Chi Minh City and ethical approval was obtained from the Human Research Ethics Committee of the Northern Territory Department of Health and Menzies School of Health Research, Australia and the Vietnam Ministry of Health Ethics Committee. The clinical trial registration number is NCT01953510.

The Vietnam Pneumococcal Project involved 1201 infants randomised at 2 months of age to one of six infant PCV schedules and 199 children recruited at 18 months of age.[14] This current study involves one of the groups enrolled at 2 months of age, who received a first dose of PCV10 at 18 months of age (PCV10 group), and the group enrolled at 18 months of age as unvaccinated controls (unvaccinated group). Participants were recruited from Districts 4 and 7 in Ho Chi Minh City, Vietnam, with the unvaccinated group recruited in parallel when the PCV10 group participants were reaching 18 months of age. Both groups were followed up to 24 months of age, at which point they received a dose of PCV10.

### 5.2 Study procedures and laboratory analyses

Blood samples were collected at 18, 19 and 24 months of age for immunogenicity assessments. A modified WHO ELISA method[19] was used to measure serotype-specific IgG levels to all PCV10 serotypes (1, 4, 5, 6B, 7F, 9V, 14, 18C, 19F and 23F), as well as the cross-reactive serotypes 6A and 19A, at all time points. Opsonophagocytic assays (OPA) for these 12 serotypes were performed in a subset of participants (the first 50 paired samples per group) at the 19 and 24 month time points, using a previously published multiplexed OPA method[13] with some minor modifications. Peripheral blood mononuclear cells (PBMCs) were isolated in a subset of participants (the first 100 recruited per group) at the 18 and 24 month time points, for measurement of pneumococcal-specific Bmem by ELISPOT for seven serotypes (1, 5, 6B, 14, 18C, 19A and 23F) using an established method.[20]

Nasopharyngeal swabs were collected at 18 and 24 months, and were stored and transported consistent with WHO guidelines.[21] Pneumococcal detection and quantification was performed using real-time quantitative PCR (qPCR) targeting the *lytA* gene. Molecular serotyping was conducted using DNA microarray as previously described.[22]

### 5.3 Outcomes

Serotype-specific IgG responses by ELISA were summarised in terms of the proportion of children with IgG ≥0•35µg/ml and the geometric mean concentrations (GMCs) of IgG. Functional antibody responses by OPA were summarised in terms of the proportion of children with an opsonisation index (OI) ≥8 and the geometric mean OIs (GMOIs). Serotype-specific Bmem levels were expressed as the mean number of antibody secreting cells (ASCs) per million PBMCs.

Pneumococcal carriage was described in terms of overall carriage (all capsular pneumococci), VT-carriage (carriage of a serotype contained in the PCV10 vaccine), non-VT-carriage (carriage of a serotype not in the PCV10 vaccine, excluding non-encapsulated pneumococci), and serotype 6A or 19A carriage. Serotype ‘11F-like’ was reported as 11A,[23] and serotypes 15B and 15C were reported as 15B/C as these serotypes interconvert.[24] Pneumococcal density (determined by *lytA* qPCR) was multiplied with the relative abundance of the serotype in the sample (determined by microarray) to determine serotype-specific density.

### 5.4 Statistical Analysis

The proportions of vaccinated and unvaccinated children with a serotype-specific IgG concentration ≥0•35µg/ml at 18 months of age, four weeks post-vaccination (19 months of age), and six months post-vaccination (24 months of age) were compared using a two-sided Fisher's exact test. The same analysis was performed for the proportion of children with OI ≥8 at the 19 and 24 month time points.

Serotype-specific IgG concentrations and OIs for all serotypes were log_10_-transformed to calculate GMCs and GMOIs with 95% CI, respectively. Groups were compared using a two-sample unpaired t-test. The mean ASCs per million PBMCs with 95% CI were compared between groups at 18 and 24 months using a two-sample unpaired t-test. A pre/post comparison of the mean ASCs were also compared between 18 and 24 months within each group using a two-sample paired t-test.

Carriage prevalence was compared between groups using a two-sided Fisher's exact test. Density data for pneumococcal carriers were log_10_-transformed and reported as log_10_ genome equivalents per ml (log_10_ GE per ml). As the transformed density data were not normally distributed, groups were compared using the non-parametric Mann-Whitney U-test.

Statistical analyses were conducted using GraphPad Prism version 7•04 (GraphPad Software, Inc.) and Stata version 14•2 (StataCorp LLC). For all comparisons, a 0.05 alpha level was used when evaluating strength of evidence for differences between groups.

### 5.5 Role of funding source

The funders of the study had no role in the study design, data collection, data analysis, data interpretation, or writing of the report. The corresponding author had full access to all the data in the study and had final responsibility for the decision to submit for publication.

## 6 RESULTS

**Figure 1:**
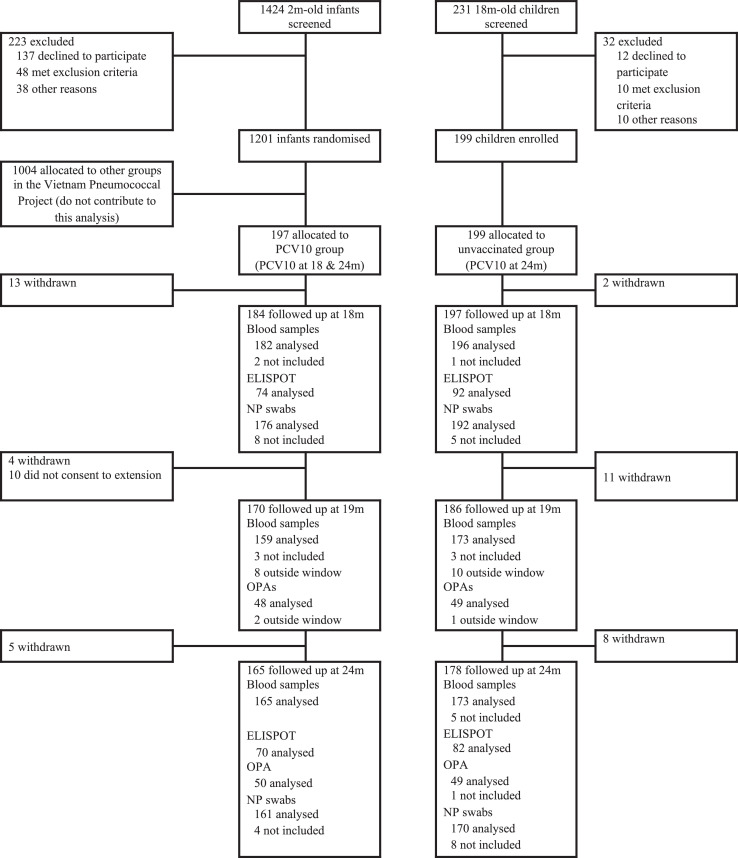
**Trial Profile.** Blood samples not included in analyses were due to no sample obtained (n=11, includes both participants who missed the study visit and participants who attended the visit but had no sample collected), insufficient volume collected (n=2) or excluded as a result of a protocol violation (PCV10 administered outside the trial, n=1). Nasopharyngeal swabs that were not included in analyses were due to insufficient DNA for microarray (n=15), no sample obtained (n=5, includes both participants who missed the study visit and participants who attended the visit but had no sample collected), pneumococcal carriage status was unable to be determined (n=3) or excluded as a result of a protocol violation (PCV10 administered outside the trial or sample collected after administration of PCV10, n=2).

Between 30 September 2013 and 9 January 2015, 197 infants were enrolled at two months of age and allocated to receive a single dose of PCV10 at 18 months of age as part of the Vietnam Pneumococcal Project. Between 14 April 2015 and 12 May 2016, 199 children were recruited at 18 months of age as an unvaccinated control group (Figure 1). Participant characteristics were similar between groups except for the median age and antibiotic use for the past fortnight at the 18 month visit (Table 1).

**Table 1:** Participant characteristics by study group

	PCV10(Group F)	Unvaccinated (Group G)	P-value*
N at enrolment	197	199	
District, 7^‡^	110/197 (55.8%)	92/199 (46.2%)	0.056
Age at enrolment (months)	2.1 (1.9-2.5)	18.3 (17.4-20.3)	NA
Sex, female	97/197 (49.2%)	86/199 (43.2%)	0.229
Birthweight (g)†	3208 (395)	3264 (423)	0.326
Hospital-based delivery†	144/197 (73.1%)	152/198 (76.8%)	0.400
Delivery by elective caesarean†	34 /196 (17.3%)	44/196 (22.4%)	0.206
Cigarette smoker in house	125/197 (63.5%)	129/199 (64.8%)	0.776
N at 18 month visit	185	197	
Age at 18 month visit (months)	18.1 (17.9-19.9)	18.3 (17.4-20.3)	<0.001
Any current breastfeeding†	21/184 (11.4%)	32/197 (16.2%)	0.173
Presence of URTI symptoms†	28/184 (15.2%)	31/197 (15.7%)	0.889
Antibiotic use in past fortnight†	20/184 (10.9%)	40/197 (20.3%)	0.012
Current antibiotic use†	10/184 (5.4%)	8/197 (4.1%)	0.528

Data are n/N (%), median (range) or mean (SD).

*P-values based on chi-squared test (for comparisons of proportions), Wilks' lambda (for comparisons of means), or quantile regression (for comparisons of medians).

† Data not available for all participants. ^‡^Participants were recruited from two districts in Ho Chi Minh City (District 4 and District 7).

**Figure 2:**
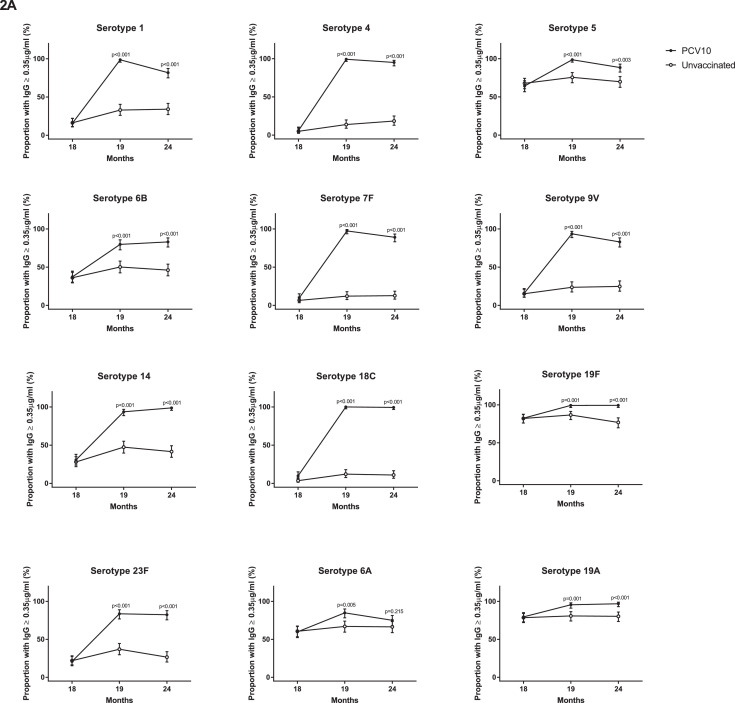

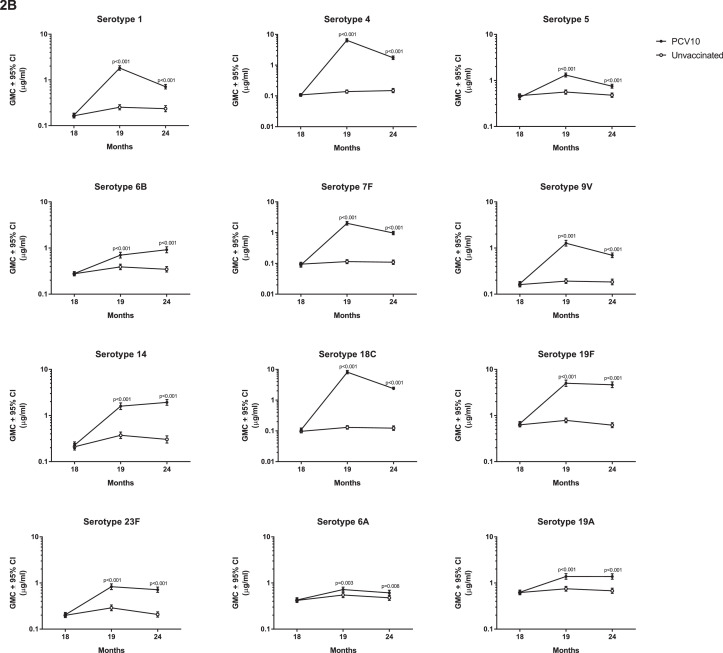
**Comparison of serotype-specific IgG responses between vaccinated and unvaccinated children.** (A) The proportion of children achieving serotype-specific IgG ≥0•35µg/mL and (B) Geometric mean concentration (GMCs) of serotype-specific IgG at 18, 19 and 24 months of children who received PCV10 at 18 months of age or who were unvaccinated. Bars represent 95% CIs. P-values were calculated using a two-sided Fisher's exact test to examine difference in proportions (A) and unpaired two-sided t-test to examine difference in GMCs (B) between groups.

**Figure 3:**
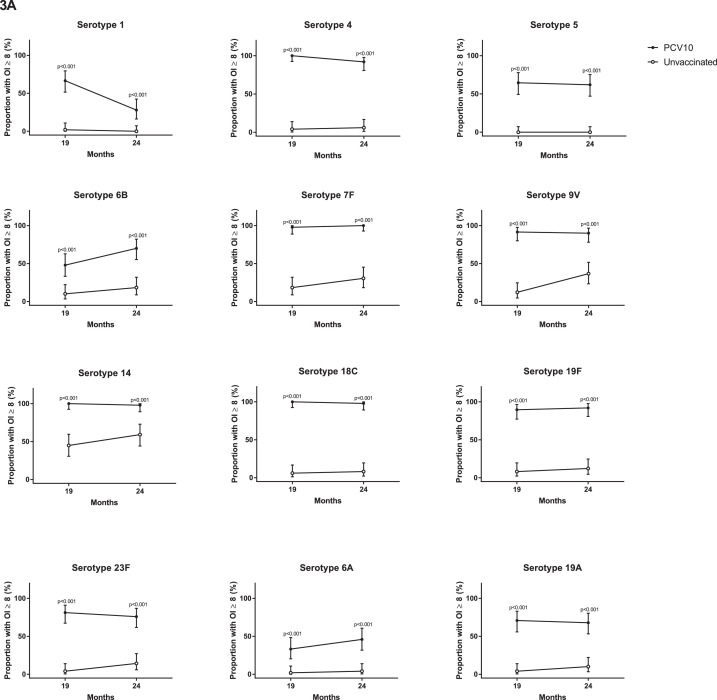

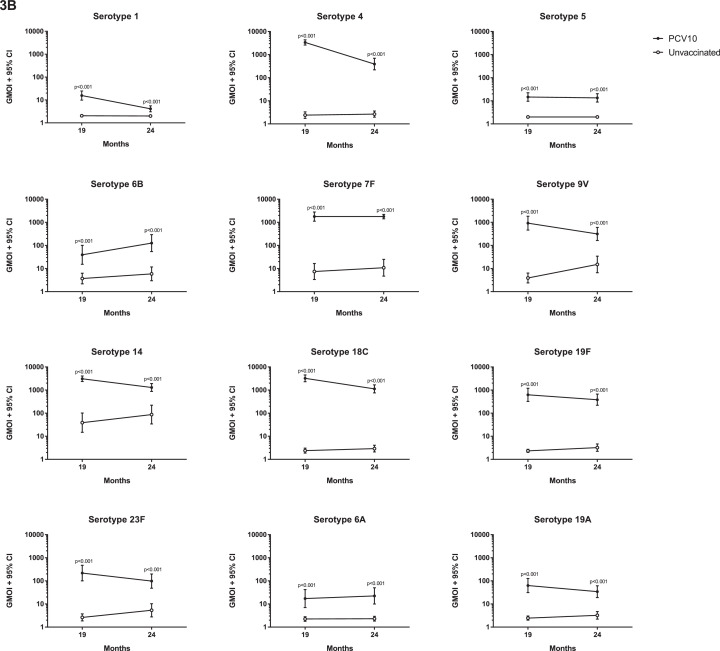
**Comparison of OPA responses between vaccinated and unvaccinated children.** (A) The proportions of children achieving opsonisation index (OI) ≥8 and (B) Geometric mean opsonisation indices (GMOIs) at 19 and 24 months of children who received PCV10 at 18 months of age or who were unvaccinated. Bars represent 95% CIs. P-values were calculated using a two-sided Fisher's exact test to examine difference in proportions (A) and an unpaired two-sided t-test to examine difference in GMOIs (B) between groups.

At 18 months of age, prior to PCV10, there were no differences in the proportion of children with serotype-specific IgG ≥0•35µg/ml or the IgG GMCs between the groups (Figure 2, Appendix Table S1). At this time, antibody levels were low for most serotypes, although over half the participants in each group had IgG ≥0•35µg/ml for serotypes 5, 19F, 6A, and 19A (Figure 2, Appendix Tables S1 and S2). At 19 months of age, four weeks after a single dose of PCV10, the proportion of children with IgG ≥0•35µg/ml and the IgG GMCs were higher in the vaccinated group than the unvaccinated group for all PCV10 and cross-reactive serotypes (Figure 2, Appendix Table S2). For eight out of ten PCV10 serotypes, 94-100% of vaccinated children had IgG levels ≥0•35µg/ml, with 80% for serotype 6B and 84% for serotype 23F. A similar trend was found for OPA, where the vaccinated group had a higher proportion of children with OI ≥8 and higher GMOIs for all 12 serotypes at 19 months of age compared with the unvaccinated group (Figure 3, Appendix Table S3).

By 24 months of age, six months after a single dose of PCV10, the proportions of children with IgG ≥0•35µg/ml were still higher in the vaccinated group compared with the unvaccinated group for all serotypes except the cross-reactive serotype 6A (Figure 2). The IgG GMCs were also higher in vaccinated children (compared with unvaccinated children) for all serotypes tested. Similarly, the proportions of children with OI ≥8 and GMOIs remained higher in the vaccinated group, compared with the unvaccinated group, for all 12 serotypes (Figure 3, Appendix Table S4).

For some serotypes, the IgG and OPA responses were not always concordant. At 19 months of age, 99% of vaccinated children had IgG ≥0•35µg/ml for serotype 1, whilst only 67% had OI ≥8 following a single dose of PCV10. This pattern was also observed for serotypes 5 and 6B, where 99% and 80% of vaccinated children had IgG ≥0•35µg/ml but only 65% and 48% of vaccinated children had OIs ≥8, respectively. This was also seen for the cross-reactive serotypes 6A in vaccinated children (85% with IgG ≥0•35µg/ml and only 33% with OIs ≥8) and to a lesser extent for serotype 19A (96% with IgG ≥0•35µg/ml and 71% with OI ≥8, Figure 2A and 3A, Appendix Tables S1 and S3).

There were no differences in the levels of pneumococcal-specific Bmem between the groups prior to vaccination at 18 months of age for any of the seven serotypes tested (Table 2). At 24 months, pneumococcal-specific Bmem were generally higher in vaccinated children compared with unvaccinated children, but this was only significant for serotypes 1 and 18C (Table 2). However, Bmem levels increased between 18 and 24 months of age in vaccinated children for all serotypes except 19A, while there were no differences in Bmem levels between 18 and 24 months in unvaccinated children.

**Table 2:** Serotype-specific memory B cell (Bmem) levels in children who received PCV10 at 18 months of age or who were unvaccinated.

	18 months			24 months			18 months vs 24 months	
	Mean of ASCs per 10^6^cultured PBMCs, % (95% CI)			Mean ASCs per 10^6^cultured PBMCs, % (95% CI)			PCV10 (Group F)	Unvaccinated (Group G)
	PCV10 (Group F, n=74)	Unvaccinated (Group G, n=92)	P-value*	PCV10 (Group F, n=70)	Unvaccinated (Group G, n=82)	P-value*	P-value†	P-value‡
PCV10 serotypes								
1	1.34(0.77 – 1.90)	1.67(1.04 – 2.31)	0.445	2.99(2.18 – 3.79)	1.48(0.88 – 2.08)	0.003	0.002	0.569
5	1.19(0.71 – 1.67)	1.73(1.09 – 2.37)	0.200	2.36(1.67 – 3.04)	1.90(1.19 – 2.61)	0.365	0.006	0.944
6B	0.82(0.48 – 1.17)	1.46(0.96 – 1.96)	0.050	1.73(1.16 – 2.30)	1.24(0.69 – 1.79)	0.227	0.008	0.928
14	1.28(0.76 – 1.81)	1.82(1.12 – 2.53)	0.240	2.94(2.13 – 3.75)	2.06(1.24 – 2.88)	0.134	0.001	0.603
18C	1.16(0.66 – 1.66)	1.92(1.30 – 2.54)	0.067	7.54(5.19 – 9.89)	2.46(1.66 – 3.27)	<0.001	<0.001	0.358
23F	1.46(0.96 – 1.96)	2.03(1.46 – 2.61)	0.148	2.43(1.76 – 3.09)	2.55(1.73 – 3.37)	0.824	0.025	0.179
Cross-reactive serotype								
19A	1.10(0.66 – 1.53)	1.83(1.24 – 2.41)	0.058	1.70(1.17 – 2.23)	1.71(1.06 – 2.35)	0.986	0.055	1.00

⁎ P-values were calculated using unpaired two-sided t-test to examine the difference between groups.

† P-values and

‡ P-values were calculated using paired two-sided t-test to examine the difference between time points for the vaccinated group and unvaccinated group, respectively.

The prevalence of capsular pneumococcal carriage was similar in both groups at 18 months of age (25% in the vaccinated group compared with 23% in the unvaccinated group; Table 3). There were also no differences in VT carriage, non-VT carriage, or carriage of serotypes 6A/19A between groups at this time point (Table 3). At 18 months of age, the most commonly carried serotypes were 19F, 23F, 6B and 6A (Appendix Table S5). At 24 months of age, VT carriage was 60% lower in the vaccinated group compared with the unvaccinated group (5.0% vs. 12.4%, Table 3). This led to a 35% reduction in all capsular pneumococcal carriage, although this did not reach statistical significance. There were no differences between groups at 24 months of age in relation to non-VT or serotype 6A/19A carriage. Pneumococcal density among carriers did not differ between groups at either 18 or 24 months of age, in terms of overall density, VT density or non-VT density (Appendix Figure S1).

**Table 3:** Pneumococcal carriage prevalence (%) at 18 and 24 months of age in children who received PCV10 at 18 months of age or who were unvaccinated.

	18 months			24 months		
	PCV10(Group F, n=176)	Unvaccinated(Group G, n=192)		PCV10(Group F, n=161)	Unvaccinated(Group G, n=170)	
	Prevalence (%)		P-value*	Prevalence (%)		P-value*
All capsular pneumococci	25.0(18.8-32.1)	22.9(17.2-29.5)	0.714	13.7(8.8-20.0)	21.2(15.3-28.1)	0.083
PCV10 carriage	13.1(8.5-19.0)	15.1(10.4-21.0)	0.654	5.0(2.2-9.6)	12.4(7.8-18.3)	0.020
Non-PCV10 carriage	13.1(8.5-19.0)	8.3(4.8-13.2)	0.175	9.9(5.8-15.6)	10.6(6.4-16.2)	0.859
6A/19A carriage	6.8(3.6-11.6)	4.2(1.8-8.0)	0.358	5.6(2.6-10.3)	6.5(3.3-11.3)	0.820

⁎ P-values were calculated using a two-sided Fisher's exact test to examine the difference in carriage prevalence between vaccinated and unvaccinated children.

## 7 DISCUSSION

There are no specific recommendations for the optimal use of PCVs during the second year of life, either for catch-up vaccination (as part of national PCV introduction) or during humanitarian crises and other emergency settings, due to a paucity of data. Here we found that a single dose of PCV10 at 18 months of age induced strong antibody responses by ELISA to all vaccine and cross-reactive serotypes, and strong functional antibody responses by OPA to most serotypes. These responses lasted for at least six months. We also found an increase in Bmem levels and a large reduction in VT carriage at 24 months in children vaccinated with a single dose of PCV10.

Previous data on responses to a single dose of PCV in the second year of life are limited. In Kenya, immune responses to two doses of PCV10 administered 2-6 months apart were investigated among children aged 12-59 months.[10] Over 90% of participants aged 12-23 months had protective levels of antibody to five serotypes after a single dose. The same was true for eight serotypes (all except 6B and 23F) among children aged 24-59 months, and GMCs were also higher in the 24-59 month group compared with the 12-23 month group for five serotypes. In a recent study in Burkina Faso, over 90% of participants had protective levels of antibody to all serotypes except 3, 6B and 23F three months following a single dose of PCV13 at 12-15 months of age.[8] Similarly, a multicentre trial of meningococcal vaccine that also measured responses to a single dose of PCV13 at 12-14 months of age found protective levels of antibody to all serotypes except serotype 3 in over 90% of participants.[9] These findings are broadly consistent with our data, in which over 90% of participants had protective levels of antibody to most serotypes. Exceptions were 6B and 23F, although lower correlates of protection have been proposed for these serotypes[25]. Together these results suggest that a single dose of PCV in the second year of life is likely to be protective for most serotypes.

A single dose of PCV10 resulted in an increase in Bmem levels between 18 and 24 months of age for all six vaccine serotypes tested. Limited data exist on Bmem levels after primary vaccination with PCVs in infants and children but those few studies that investigated the effect of PCVs on Bmem also show an increase in levels after vaccination[20,[26], [27], [28]]. From other groups within the Vietnam Pneumococcal Project, a primary series vaccination of PCV10 or PCV13 induced high levels of serotype-specific Bmem.[27] Another study that compared the effect of a single dose of PCV7 in naïve adults and toddlers (aged 12 months) found a significant increase in memory B cells by day seven post-vaccination in both groups.[26] In Kenyan toddlers 12-23 months of age, a single dose of PCV10 generated higher Bmem one month after vaccination for serotypes 1 and 19F.[29] Although we did not assess Bmem levels at their peak (seven days post-vaccination), we were still able to observe an increase in Bmem numbers after six months for all vaccine serotypes tested in the PCV10 group that was not seen in the unvaccinated group. This suggests that a single dose of PCV10 induces immunological memory responses that are likely to be protective for up to six months post-vaccination.

A single dose of PCV10 at 18 months led to a dramatic 60% reduction in VT carriage at 24 months of age compared with unvaccinated controls. This is consistent with the Kenya trial in which children aged 12-59 months who received one dose of PCV10 had approximately 30% lower VT carriage compared with unvaccinated controls after six months, although this was not statistically significant.[10] Children between the ages of 12 and 36 months have been shown to be the key transmitters of pneumococci;[30,31] any reduction in carriage in this age group therefore has the potential to generate significant herd protection. Whether a single PCV dose would have a similar impact in settings where carriage and/or disease rates are higher is unknown and should be addressed in future studies. However, in situations where only one dose can only be given (emergency settings or catch-up campaigns), a single dose of PCV is likely to be of benefit.

The use of both ELISA and OPA assays in this study revealed a discordance in the responses to some serotypes. Serotypes 1, 5, 6B and the cross-reactive serotype 6A were all poorly immunogenic by OPA, similar to previous studies.[5,32] We have previously shown that poor OPA responses to serotype 1 post-primary series can occur despite strong ELISA responses, although this was corrected following a booster dose.[32] This is consistent with the Kenya trial, which found that a second dose of PCV10 was required to bring OI ≥8 above 80% for serotypes 1, 5, 6B, 14 and 19F. As OPA may be a better marker of protection, a second dose might be considered to improve the disconnect between IgG and OPA responses, such as in African settings where serotype 1 disease is high.

The high cost associated with PCVs remains a major impediment for their use in humanitarian immunisation programs.[11] Based on our findings, a single dose of PCV10 at 18 months could be effective during emergency situations to protect children living in extremely vulnerable conditions where multi-dose vaccination is not feasible. Such an approach has been successfully used with measles supplementary immunisation campaigns, wherein expanded target-age groups allowed for catch-up immunisations and a reduced dosing schedule.[33] Our findings also suggest that a single dose of PCV10 in the second year of life could also be considered for catch-up vaccination programs as part of infant PCV introduction. As the interest in reduced-dose PCV schedules increases following the recent introduction of a 1+1 schedule in the UK[34], the role of catch-up programs is likely to become increasingly important for maximising the impact of vaccine introduction. Catch-up campaigns are also crucial for extending protection to older children who miss doses due to interruptions in routine immunisation such as experienced in many countries due to the current COVID-19 pandemic.

A limitation of this study was that the two groups were not recruited at the same time. However, they were recruited from the same study sites, and few differences in participant characteristics between groups were observed at the 18 month visit. Antibiotic use in the past fortnight was reported almost twice as frequently in the unvaccinated group than the PCV10 group, although reported current antibiotic use was similar between groups. Any effect of increased antibiotic use in the unvaccinated group would be to reduce the pneumococcal carriage rates and thus bias the results towards no effect. Importantly, the immunological and carriage data at 18 months of age did not differ between groups, and so the differences in participant characteristics are unlikely to have affected our results.

In conclusion, a single dose of PCV10 at 18 months of age was highly immunogenic and produced functional immunity that was still detectable at 24 months of age for all vaccine serotypes. It was also able to induce immunological memory and reduced VT carriage in vaccinated children at 24 months of age. Together, these data support the use of a single dose of PCV10 in the second year of life for catch-up vaccination and in emergency situations to provide both individual and herd protection.

### 7.1 Data sharing statement

The study protocol and informed consent form have been published and are freely available.[14] Data will be made publicly available in accordance with the rules set out by the Bill & Melinda Gates Foundation.

## 8 Contributors

RAH was responsible for the OPA studies, performed the data analysis and wrote the first draft of this manuscript with input from BT and PVL. PVL contributed to the trial design and oversaw the immunology. CS contributed to the trial design and oversaw the microbiology, with MLN, BDO, HSV and JH. BT was involved with the design and day-to-day management of the trial and contributed to the data analysis. KB, NTT, and DYU were involved in the design, establishment, day-to-day management, and implementation of the trial. VTTD managed and performed laboratory testing at the Pasteur Institute laboratory, with TVP also contributing to laboratory testing. LS contributed to the OPA studies. ZQT contributed to the B cell studies. CDN and YBC advised on the statistical analyses. AB contributed to the study design and oversaw the immunology procedures. TNH was involved in the design and establishment of the trial and had overall responsibility for the conduct of the trial in Vietnam as site principal investigator. KM conceived the study, provided oversight for the conduct of the trial and data analysis, and had overall responsibility for all aspects of the trial as the principal investigator. All authors contributed to refinement of and approved the submitted manuscript.

## Declaration of Competing Interest

Funding for this study was provided by grants from National Health and Medical Research Council of Australia (NHMRC) and/or Bill & Melinda Gates Foundation grants. Non-financial support (in the form of PCV10 vaccine doses) and funding for opsonophagocytic assays were provided by GSK Biologicals SA. KM, CS and CDN have received grant funding for a collaborative study on PCV impact on adult pneumonia from Pfizer. None of the authors have any other competing interests to declare.

## Appendix Supplementary materials

Image, application 1

Image, application 2

Image, application 3

Image, application 4
