# Supplementary material for: IMMUNOGENICITY AND IMPACT ON NASOPHARYNGEAL CARRIAGE OF A SINGLE DOSE OF PCV10 GIVEN TO VIETNAMESE CHILDREN AT 18 MONTHS OF AGE
Source: Lancet Reg Health West Pac. 2021 Sep 20;16:100273. doi: 10.1016/j.lanwpc.2021.100273 (PMC8453212; doi:10.1016/j.lanwpc.2021.100273)
Supplement: Supplementary file 1 [file mmc1.docx]

**Supplementary File - List of captions**

**Figure S1: Pneumococcal carriage density among pneumococcal carriers a) at 18 months of age and b) at 24 months of age.** Median (IQR) density (log10 genome equivalents per ml) of capsular, PCV10-type and non-PCV10-type among pneumococcal carriers at a) 18 months of age and b) 24 months of age who received a single dose of PCV10 at 18 months of age or who were unvaccinated. IQR = interquartile range. PCV = pneumococcal conjugate vaccine. PCV10 = ten-valent PCV. IQR = inter-quartile range. ● denotes a datapoint greater than the 75th percentile plus 1·5 times the IQR.
