# Supplementary material for: IMMUNOGENICITY AND IMPACT ON NASOPHARYNGEAL CARRIAGE OF A SINGLE DOSE OF PCV10 GIVEN TO VIETNAMESE CHILDREN AT 18 MONTHS OF AGE
Source: Lancet Reg Health West Pac. 2021 Sep 20;16:100273. doi: 10.1016/j.lanwpc.2021.100273 (PMC8453212; doi:10.1016/j.lanwpc.2021.100273)
Supplement: Supplementary file 2 [file mmc2.docx]

**LIST OF APPENDIX TABLES AND FIGURES**

[**Table S1: Proportion (%) and 95% CI of children with IgG concentration ≥0·35µg/mL at the 18, 19, and 24 month time points** 2](#_Toc79056414)

[**Table S2: Serotype-specific IgG geometric mean concentrations (GMCs) and 95% CI of children at the 18, 19, and 24 month time points** 3](#_Toc79056415)

[**Table S3: Proportion (%) and 95% CI of children achieving opsonisation index (OI) >8 at the 18, 19, and 24 month time points** 4](#_Toc79056416)

[**Table S4: Geometric mean opsonisation indices (GMOIs) and 95% CI of children at the 18, 19, and 24 month time points** 5](#_Toc79056417)

[**Table S5: Serotype-specific carriage prevalence at 18 and 24 months of age, % (n)** 6](#_Toc79056418)

[**Figure S1: Pneumococcal carriage density among pneumococcal carriers a) at 18 months of age and b) at 24 months of age** 7](#_Toc79056419)

# **Table S1: Proportion (%) and 95% CI of children with IgG concentration ≥0·35µg/mL at the 18, 19, and 24 month time points**

|  | **18 months** | | |  | **19 months** | | |  | **24 months** | | |
| --- | --- | --- | --- | --- | --- | --- | --- | --- | --- | --- | --- |
|  | PCV10  (n=182) | Unvaccinated (n=196) | P value* |  | PCV10  (n=159) | Unvaccinated (n=173) | P value* |  | PCV10  (n=165) | Unvaccinated (n=173) | P value* |
| PCV10 serotypes |  |  |  |  |  |  |  |  |  |  |  |
| 1 | 15·9  (10·9 to 22·1) | 16·3  (11·4 to 22·3) | >0·999 |  | 98·7  (95·5 to 99·8) | 32·9  (26·0 to 40·5) | <0·001 |  | 81·8  (75·1 to 87·4) | 34·1  (27·1 to 41·7) | <0·001 |
| 4 | 6·0  (3·1 to 10·6) | 5·1  (2·5 to 9·2) | >0·999 |  | 99·4  (96·5 to 100) | 13·9  (9·1 to 19·9) | <0·001 |  | 95·2  (90·7 to 97·9) | 18·5  (13·0 to 25·1) | <0·001 |
| 5 | 64·3  (56·9 to 71·2) | 67·9  (60·8 to 74·3) | 0·655 |  | 98·7  (95·5 to 99·8) | 75·7  (68·6 to 81·9) | <0·001 |  | 88·5  (82·6 to 92·9) | 69·9  (62·5 to 76·7) | 0·003 |
| 6B | 37·4  (30·3 to 44·8) | 36·2  (29·5 to 43·4) | >0·999 |  | 79·9  (72·8 to 85·8) | 50·3  (42·6 to 58·0) | <0·001 |  | 83·0  (76·4 to 88·4) | 46·2  (38·6 to 54·0) | <0·001 |
| 7F | 9·9  (6·0 to 15·2) | 6·6  (3·6 to 11·1) | 0·613 |  | 97·5  (93·7 to 99·3) | 12·1  (7·7 to 18·0) | <0·001 |  | 89·1  (83·3 to 93·4) | 12·7  (8·1 to 18·6) | <0·001 |
| 9V | 15·9  (10·9 to 22·1) | 15·3  (10·6 to 21·1) | >0·999 |  | 93·7  (88·7 to 96·9) | 23·7  (17·6 to 30·7) | <0·001 |  | 83·0  (76·4 to 88·4) | 24·9  (18·6 to 32·0) | <0·001 |
| 14 | 30·8  (24·2 to 38·0) | 28·1  (21·9 to 34·9) | 0·757 |  | 93·7  (88·7 to 96·9) | 47·4  (39·8 to 55·1) | <0·001 |  | 98·8  (95·7 to 99·9) | 41·6  (34·2 to 49·3) | <0·001 |
| 18C | 9·9  (6·0 to 15·2) | 3·6  (1·4 to 7·2) | 0·164 |  | 100  (97·7 to 100) | 12·1  (7·7 to 18·0) | <0·001 |  | 99·4  (96·7 to 100) | 11·0  (6·7 to 16·6) | <0·001 |
| 19F | 82·4  (76·1 to 87·7) | 82·1  (76·1 to 87·2) | >0·999 |  | 99·4  (96·5 to 100) | 86·7  (80·7 to 91·4) | 0·001 |  | 99·4  (96·7 to 100) | 76·9  (69·9 to 82·9) | <0·001 |
| 23F | 20·9  (15·2 to 27·5) | 21·9  (16·4 to 28·4) | >0·999 |  | 83·6  (77·0 to 89·0) | 37·0  (29·8 to 44·7) | <0·001 |  | 82·4  (75·7 to 87·9) | 26·6  (20·2 to 33·8) | <0·001 |
| Cross-reactive serotypes | |  |  |  |  |  |  |  |  |  |  |
| 6A | 59·9  (52·4 to 67·1) | 60·7  (53·5 to 67·6) | >0·999 |  | 84·9  (78·4 to 90·1) | 67·1  (59·5 to 74·0) | 0·005 |  | 75·2  (67·8 to 81·5) | 66·5  (58·9 to 73·5) | 0·215 |
| 19A | 79·7  (73·1 to 85·3) | 78·6  (72·2 to 84·1) | >0·999 |  | 95·6  (91·1 to 98·2) | 80·9  (74·3 to 86·5) | 0·001 |  | 97·0  (93·1 to 99·0) | 80·3  (73·6 to 86·0) | <0·001 |

* P values were calculated using a two-sided Fisher’s exact test to examine the difference in proportions between vaccinated and unvaccinated children

# **Table S2: Serotype-specific IgG geometric mean concentrations (GMCs) and 95% CI of children at the 18, 19, and 24 month time points**

|  | **18 months** | | |  | **19 months** | | |  | **24 months** | | |
| --- | --- | --- | --- | --- | --- | --- | --- | --- | --- | --- | --- |
|  | PCV10  (n=182) | Unvaccinated (n=196) | P value* |  | PCV10  (n=159) | Unvaccinated (n=173) | P value* |  | PCV10  (n=165) | Unvaccinated (n=173) | P value* |
| PCV10 serotypes |  |  |  |  |  |  |  |  |  |  |  |
| 1 | 0·17  (0·15 to 0·19) | 0·16  (0·15 to 0·18) | 0·686 |  | 1·82  (1·62 to 2·04) | 0·25  (0·22 to 0·29) | <0·001 |  | 0·72  (0·63 to 0·80) | 0·23  (0·20 to 0·27) | <0·001 |
| 4 | 0·11  (0·10 to 0·11) | 0·11  (0·10 to 0·12) | 0·825 |  | 6·44  (5·70 to 7·27) | 0·14  (0·13 to 0·16) | <0·001 |  | 1·74  (1·53 to 1·99) | 0·15  (0·13 to 0·17) | <0·001 |
| 5 | 0·43  (0·39 to 0·48) | 0·47  (0·43 to 0·51) | 0·211 |  | 1·31  (1·19 to 1·45) | 0·56  (0·50 to 0·62) | <0·001 |  | 0·75  (0·68 to 0·83) | 0·48  (0·43 to 0·53) | <0·001 |
| 6B | 0·28  (0·25 to 0·31) | 0·28  (0·25 to 0·31) | 0·921 |  | 0·70  (0·61 to 0·81) | 0·39  (0·34 to 0·45) | <0·001 |  | 0·92  (0·79 to 1·06) | 0·35  (0·30 to 0·40) | <0·001 |
| 7F | 0·09  (0·08 to 0·10) | 0·10  (0·08 to 0·11) | 0·561 |  | 2·01  (1·76 to 2·31) | 0·11  (0·10 to 0·13) | <0·001 |  | 0·97  (0·86 to 1·10) | 0·11  (0·09 to 0·13) | <0·001 |
| 9V | 0·17  (0·15 to 0·19) | 0·16  (0·14 to 0·18) | 0·593 |  | 1·27  (1·10 to 1·46) | 0·20  (0·17 to 0·22) | <0·001 |  | 0·70  (0·62 to 0·78) | 0·18  (0·16 to 0·21) | <0·001 |
| 14 | 0·23  (0·19 to 0·27) | 0·21  (0·18 to 0·25) | 0·511 |  | 1·59  (1·35 to 1·87) | 0·37  (0·32 to 0·44) | <0·001 |  | 1·92  (1·67 to 2·21) | 0·30  (0·25 to 0·36) | <0·001 |
| 18C | 0·11  (0·10 to 0·12) | 0·10  (0·09 to 0·11) | 0·147 |  | 8·11  (7·17 to 9·18) | 0·13  (0·11 to 0·15) | <0·001 |  | 2·37  (2·14 to 2·62) | 0·12  (0·10 to 0·14) | <0·001 |
| 19F | 0·67  (0·60 to 0·74) | 0·63  (0·57 to 0·69) | 0·430 |  | 5·01  (4·28 to 5·86) | 0·78  (0·70 to 0·88) | <0·001 |  | 4·63  (4·02 to 5·34) | 0·62  (0·55 to 0·70) | <0·001 |
| 23F | 0·21  (0·18 to 0·23) | 0·20  (0·18 to 0·22) | 0·722 |  | 0·83  (0·72 to 0·96) | 0·29  (0·25 to 0·33) | <0·001 |  | 0·72  (0·63 to 0·82) | 0·21  (0·18 to 0·24) | <0·001 |
| Cross-reactive serotypes | |  |  |  |  |  |  |  |  |  |  |
| 6A | 0·43  (0·38 to 0·48) | 0·42  (0·38 to 0·46) | 0·775 |  | 0·72  (0·64 to 0·81) | 0·55  (0·48 to 0·63) | 0·003 |  | 0·61  (0·54 to 0·69) | 0·48  (0·42 to 0·54) | 0·008 |
| 19A | 0·63  (0·56 to 0·70) | 0·62  (0·56 to 0·69) | 0·868 |  | 1·38  (1·21 to 1·59) | 0·75  (0·66 to 0·84) | <0·001 |  | 1·38  (1·20 to 1·58) | 0·68  (0·60 to 0·77) | <0·001 |

* P values were calculated using a two-sided unpaired t-test to examine the difference in GMCs between vaccinated and unvaccinated children

# **Table S3: Proportion (%) and 95% CI of children achieving opsonisation index (OI) >8 at the 18, 19, and 24 month time points**

|  | **19 months** | | |  | **24 months** | | |
| --- | --- | --- | --- | --- | --- | --- | --- |
|  | PCV10  (n=48) | Unvaccinated (n=49) | P value* |  | PCV10  (n=50) | Unvaccinated (n=49) | P value* |
| PCV10 serotypes |  |  |  |  |  |  |  |
| 1 | 66·7  (51·6 to 79·6) | 2·0  (0·1 to 10·9) | <0·001 |  | 28·0  (16·2 to 42·5) | 0·0  (0·0 to 7·3) | <0·001 |
| 4 | 100  (92·6 to 100) | 4·1  (0·5 to 14·0) | <0·001 |  | 92·0  (80·8 to 97·8) | 6·1  (1·3 to 16·9) | <0·001 |
| 5 | 64·6  (49·5 to 77·8) | 0·0  (0·0 to 7·3) | <0·001 |  | 62·0  (47·2 to 75·3) | 0·0  (0·0 to 7·3) | <0·001 |
| 6B | 47·9  (33·3 to 62·8) | 10·2  (3·40 to 22·2) | <0·001 |  | 70  (55·4 to 82·1) | 18·4  (8·8 to 32·0) | <0·001 |
| 7F | 97·9  (88·9 to 99·9) | 18·4  (8·8 to 32·0) | <0·001 |  | 100  (92·9 to 100) | 30·6  (18·3 to 45·4) | <0·001 |
| 9V | 91·7  (80·0 to 97·7) | 12·2  (4·6 to 24·8) | <0·001 |  | 90·0  (78·2 to 96·7) | 36·7  (23·4 to 51·7) | <0·001 |
| 14 | 100  (92·6 to 100) | 44·9  (30·7 to 59·8) | <0·001 |  | 98·0  (89·4 to 99·9) | 59·2  (44·2 to 73·0) | <0·001 |
| 18C | 100  (92·6 to 100) | 6·1  (1·3 to 16·9) | <0·001 |  | 98  (89·4 to 99·9) | 8·2  (2·3 to 19·6) | <0·001 |
| 19F | 89·6  (77·3 to 96·5) | 8·2  (2·3 to 19·6) | <0·001 |  | 92·0  (80·8 to 97·8) | 12·2  (4·6 to 24·8) | <0·001 |
| 23F | 81·3  (67·4 to 91·1) | 4·1  (0·5 to 14·0) | <0·001 |  | 76·0  (61·8 to 86·9) | 14·3  (5·9 to 27·2) | <0·001 |
| Cross-reactive serotypes |  |  |  |  |  |  |  |
| 6A | 33·3  (20·4 to 48·4) | 2·0  (0·1 to 10·9) | <0·001 |  | 46·0  (31·8 to 60·7) | 4·1  (0·5 to 14·0) | <0·001 |
| 19A | 70·8  (55·9 to 83·0) | 4·1  (0·5 to 14·0) | <0·001 |  | 68·0  (53·3 to 80·5) | 10·2  (3·4 to 22·2) | <0·001 |

* P values were calculated using a two-sided Fisher’s exact test to examine the difference in proportions between vaccinated and unvaccinated children

# **Table S4: Geometric mean opsonisation indices (GMOIs) and 95% CI of children at the 18, 19, and 24 month time points**

|  | **19 months** | | |  | **24 months** | | |
| --- | --- | --- | --- | --- | --- | --- | --- |
|  | PCV10  (n=48) | Unvaccinated (n=49) | P value* |  | PCV10  (n=50) | Unvaccinated (n=49) | P value* |
| PCV10 serotypes |  |  |  |  |  |  |  |
| 1 | 16  (10 to 25) | 2  (2 to 2) | <0·001 |  | 4  (3 to 6) | 2  (2 to 2) | <0·001 |
| 4 | 3376  (2605 to 4375) | 2  (2 to 3) | <0·001 |  | 391  (220 to 695) | 3  (2 to 4) | <0·001 |
| 5 | 15  (10 to 22) | 2  (2 to 2) | <0·001 |  | 13  (9 to 20) | 2  (2 to 2) | <0·001 |
| 6B | 39  (15 to 101) | 4  (2 to 6) | <0·001 |  | 127  (54·5 to 295) | 6  (3 to 12) | <0·001 |
| 7F | 1779  (1127 to 2809) | 7  (3 to 17) | <0·001 |  | 1781  (1428 to 2221) | 11  (5 to 25) | <0·001 |
| 9V | 920  (461 to 1837) | 4  (2 to 6) | <0·001 |  | 314  (164 to 603) | 15  (7 to 34) | <0·001 |
| 14 | 3090  (2349 to 4065) | 39  (15 to 101) | <0·001 |  | 1295  (874 to 1919) | 87  (34 to 222) | <0·001 |
| 18C | 3225  (2303 to 4517) | 2  (2 to 3) | <0·001 |  | 1125  (753 to 1680) | 3  (2 to 4) | <0·001 |
| 19F | 622  (322 to 1200) | 2  (2 to 3) | <0·001 |  | 383  (219 to 668) | 3  (2 to 5) | <0·001 |
| 23F | 218  (100 to 474) | 3  (2 to 4) | <0·001 |  | 98  (48 to 200) | 5  (3 to 10) | <0·001 |
| Cross-reactive serotypes |  |  |  |  |  |  |  |
| 6A | 17  (7 to 42) | 2  (2 to 3) | <0·001 |  | 22  (10 to 50) | 2  (2 to 3) | <0·001 |
| 19A | 63  (31 to 127) | 2  (2 to 3) | <0·001 |  | 34  (19 to 62) | 3  (2 to 5) | <0·001 |

* P values were calculated using a two-sided unpaired t-test to examine the difference in GMOIs between vaccinated and unvaccinated children

# **Table S5: Serotype-specific carriage prevalence at 18 and 24 months of age, % (n)**

|  | **18 months** | |  | **24 months** | |
| --- | --- | --- | --- | --- | --- |
|  | PCV10  (n=176) | Unvaccinated  (n=192) |  | PCV10  (n=161) | Unvaccinated  (n=170) |
| PCV10 serotypes* |  |  |  |  |  |
| 6B | 4.0 (7) | 4.2 (8) |  | 1.2 (2) | 5.3 (9) |
| 14 | 2.3 (4) | 1.6 (3) |  | 1.9 (3) | 2.4 (4) |
| 19F | 4.0 (7) | 5.2 (10) |  | 1.9 (3) | 2.9 (5) |
| 23F | 3.4 (6) | 5.2 (10) |  | 1.2 (2) | 3.5 (6) |
| Cross-reactive serotypes |  |  |  |  |  |
| 6A | 5.7 (10) | 2.6 (5) |  | 3.1 (5) | 3.5 (6) |
| 19A | 1.1 (2) | 1.6 (3) |  | 2.5 (4) | 2.9 (5) |
| Non-vaccine serotypes |  |  |  |  |  |
| 15A | 2.3 (4) | 1.0 (2) |  | 1.2 (2) | 1.2 (2) |
| 15BC | 0.0 (0) | 1.0 (2) |  | 1.2 (2) | 1.2 (2) |
| 23A | 2.3 (4) | 0.0 (0) |  | 1.9 (3) | 2.4 (4) |
| Other† | 1.7 (3) | 2.1 (4) |  | 1.2 (2) | 0.6 (1) |

* There were no instances of carriage of the other PCV10 serotypes (1, 4, 5, 7F, 9V, or 18C). † The 10 other non-vaccine serotypes comprised: 4 x serotype 34 (2 from each group at 18 months), and 1 each of serotypes 3 (vaccinated group at 18 months), 6C (vaccinated group at 24 months), 7C (vaccinated group at 24 months), 11A (unvaccinated group at 18 months), 13 (unvaccinated group at 18 months), and 35B (unvaccinated group at 24 months).

# **Figure S1: Pneumococcal carriage density among pneumococcal carriers a) at 18 months of age and b) at 24 months of age**

**a) 18 months of age b) 24 months of age**


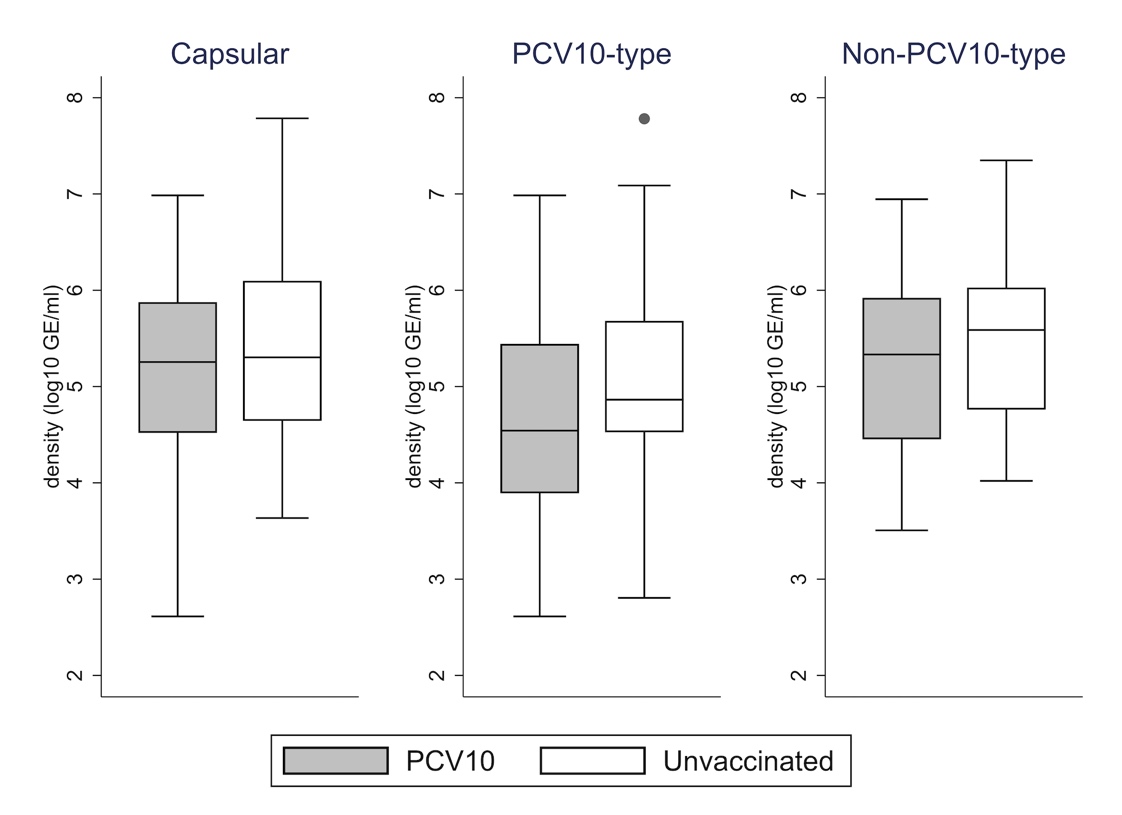

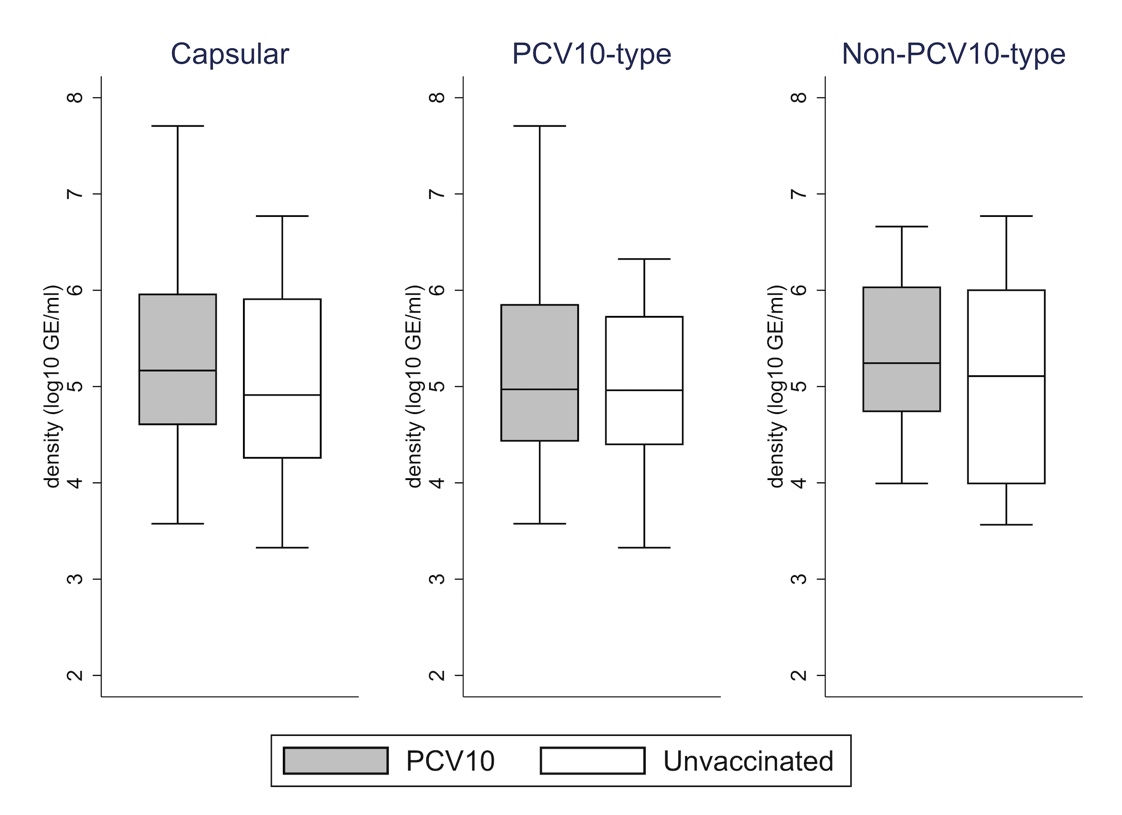


Median (IQR) density (log10 genome equivalents per ml) of capsular, PCV10-type and non-PCV10-type among pneumococcal carriers at a) 18 months of age and b) 24 months of age who received a single dose of PCV10 at 18 months of age or who were unvaccinated. IQR = interquartile range. PCV = pneumococcal conjugate vaccine. PCV10 = ten-valent PCV. IQR = inter-quartile range. ● denotes a datapoint greater than the 75th percentile plus 1·5 times the IQR.
